# A new validated facile HPLC analysis method to determine methylprednisolone including its derivatives and practical application

**DOI:** 10.1038/s41598-023-38539-2

**Published:** 2023-07-17

**Authors:** Mostafa F. Al-Hakkani

**Affiliations:** 1 Department of Chemistry, Faculty of Science, Al-Azhar University, Assiut Branch, Assiut, 71524 Egypt; 2 Department of Research, Development, and Stability, UP Pharma, Industrial Zone, Arab El Awamer, Abnoub, 76, Assiut, Egypt

**Keywords:** Analytical chemistry, Inorganic chemistry, Medicinal chemistry

## Abstract

Methylprednisolone sodium succinate (MPSS) is a parenteral water-soluble corticosteroid ester. It gives three peaks methylprednisolone (MP), 17-methylprednisolone hemisuccinate (17-MPHS), and methylprednisolone hemisuccinate (MPHS) that share in the assay determination as total MP. It is used on a wide scale in prescribed anti-inflammatory drugs as a common use. The current study aimed to find a rapid RP-HPLC method of MP and its derivatives analysis with high linearity, repeatability, sensitivity, selectivity, and inexpensive to use without the need for any special chemical reagents. The use of the current method achieved a satisfactory result to detect, determine and separate the MP, 17-MPHS, and MPHS in a short time. The chromatographic system consists of RP-HPLC using the BDS column (250 mm × 4.6 mm × 5 μm). The mobile phase was prepared by mixing the WFI: glacial acetic acid: acetonitrile in a volume ratio (63:2:35) at a flow rate of 2.0 mL/min with detection wavelength at 254 nm at room temperature and injection volume 20 μL. The method manifested a satisfied linearity regression R^2^ (0.9998–0.99999) with LOD 143.97 ng/mL and 4.49 µg/mL; and LOQ 436.27 ng/mL and 13.61 µg/mL for MP and MPHS respectively. The method proved its efficiency via system suitability achievement in the robustness and ruggedness conduction according to the validation guidelines. High sensitivity according to its LOD and LOQ. So, the current method could be considered in the pharmaceutical industry. The suggested method has been successfully implemented in the Egyptian local market for the quantitative assessment of the assay of the finished product.

## Introductıon

**Figure 1:**
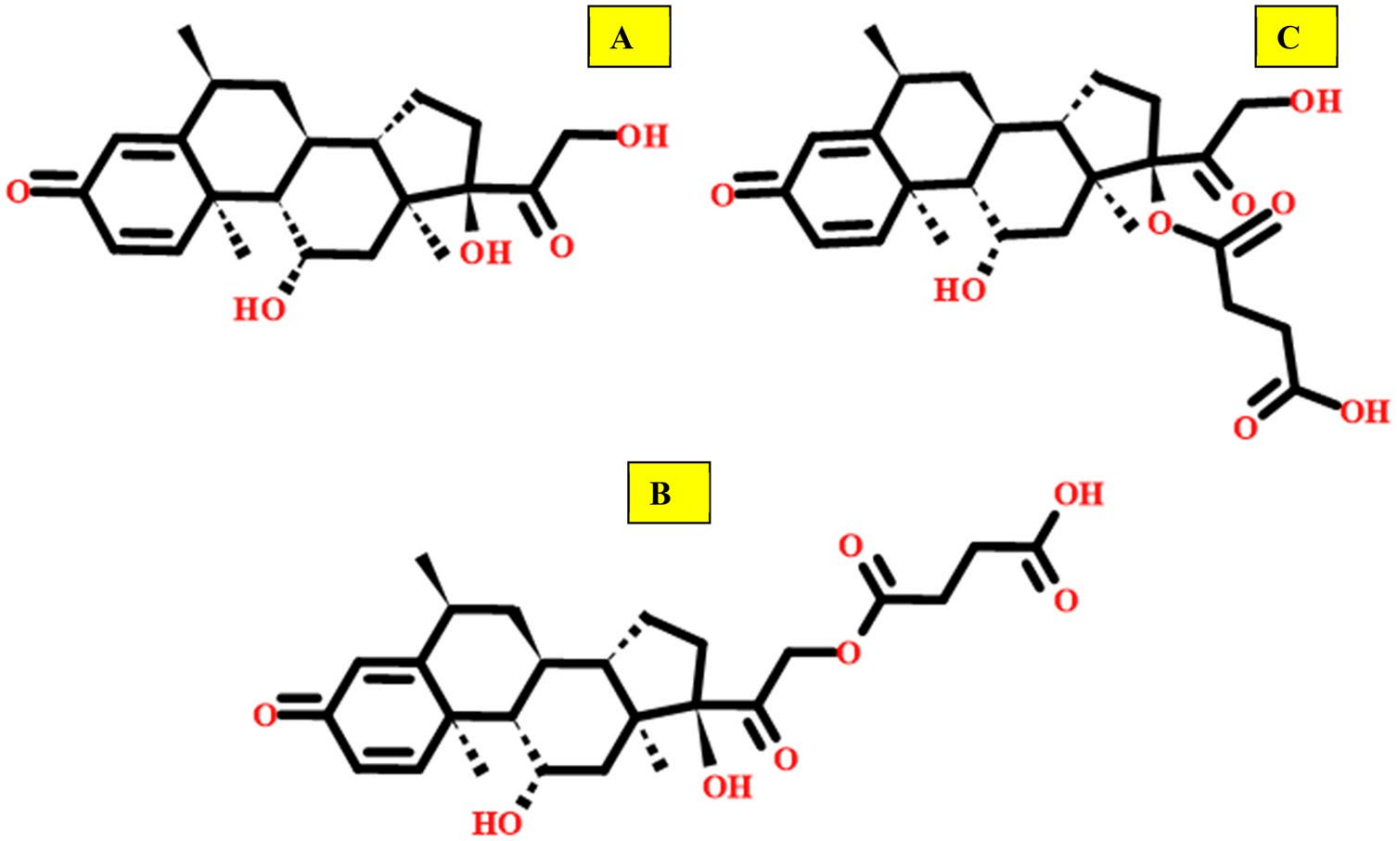
Structure of (**A**) methylprednisolone [MP], (**B**) methylprednisolone hemisuccinate [MPHS], (**C**) methylprednisolone 17-hemisuccinate [17-MPHS].

Methylprednisolone sodium succinate (MPSS) is the sodium salt of methylprednisolone hemisuccinate (MPHS). The IUPAC name of MPHS is 4-[2-[(6S,8S,9S,10R,11S,13S,14S,17R)-11,17-dihydroxy-6,10,13-trimethyl-3-oxo-7,8,9,11,12,14,15,16-octahydro-6H-cyclopenta[a]phenanthren-17-yl]-2-oxoethoxy]-4-oxobutanoic acid (Fig. 1). MPSS is the water-soluble corticosteroid ester of methylprednisolone and it is used for the treatment of cardiac, severe allergic reactions, hypoxic emergencies, respiratory diseases, ophthalmic diseases, dermatologic diseases, antineoplastic, hormonal, anti-inflammatory, neoplastic diseases, hematological disorders, nervous system conditions, and endocrine disorders. MPSS has the same anti-inflammatory and metabolism effects as methylprednisolone (MP) when administered parenterally and also at equal quantities, the two molecules have the same biologic action^1^.

The wide spectrum of MPSS drug makes it important in the field of pharmaceutical trade, which necessitates the need to find effective, simple, easy, and rapid methods for assay determination. In addition, a sensitive method should be conducted at low concentrations of this drug preparation, when this method is used to estimate MPSS after washing cleaning machines and production lines. The sensitive method should be conducted to ensure the effectiveness of the cleaning method to remove the drug residual effects of this drug that may be entered into the next product in the production process, causing a completely unacceptable cross-contamination process. This type of contamination is according to the quality standards mentioned in the rules of good manufacturing practice^2–4^.

There are many different methods with more than one technique in the analysis tools being conducted for the assay determination of MP, including flow injection analysis with LC-Q-TOF MS^5^, HPLC–MS^1^, RP-HPLC^6–9^, voltammetric techniques^10^, SWNTs/EPPGE^11^, spectrophotometrically^12^.

However, HPLC–UV detection is an easy, accurate, and inexpensive method, both at an academic and commercial level rate. The United States-Pharmacopoeia (USP44-NF 39 2021)^13^ issued the analysis method for determining MP, MPHS. The mobile phase is composed of Butyl chloride, water-saturated butyl chloride, tetrahydrofuran, methanol, and glacial acetic acid in the ratio (95:95:14:7:6) with a stationary phase column of 3.9-mm × 30-cm; packing L3 at a flow rate of about 1.0 mL per minute. Also, the standard and test should be dissolved in a diluent of chloroform and glacial acetic acid (97:3) using the Fluorometholone as internal standard. The retention time is about 25 min of MPHS.

Most of the MP and its derivatives for MPHS and 17-MPHS conducted an HPLC analysis method using a high percentage of the organic modifiers from methanol, acetonitrile, special reagents such as chloroform, tetrahydrofuran, butyl chloride, tetrabutylammonium hydroxide, adjusted pH buffer solutions, gradient program^7,8,14–17^, special type of separation HPLC column^6^, using guard column cartridge^14^. Additionally, the separation process is a time consumed. Also, some methods used a high flow rate of 4.0 mL/min and a special column as Zorbax Eclipse XDB-C18 (250 mm × 9.4 mm; 5 μm)^1^. These factors are used to get the optimum peak shape with ideal tailing^18,19^.

The field of scientific research has recently tended to purify industrial wastewater, pharmaceutical factories, and hospitals, especially for antibiotics. So, finding easy, fast, accurate, and economical methods has become an urgent necessity^2–4^.

In this manuscript, we discuss a suggested method using a simple, rapid, and robust methodological approach for the detection and evaluation of both the methylprednisolone drug and its derivatives. Additionally, the analysis method functions under simple chemical conditions and is easily accessible to any general laboratory. An analytical comparison of the determination of methylprednisolone employing various methods was also done.

## Materials and methods

Methyl Prednisolone reference standard (MP), Methyl Prednisolone hemisuccinate reference standard (MPHS), and Methylprednisolone sodium succinate working standard (MPSS) was supplied by UP pharma (Assuit, Egypt). Acetonitrile HPLC-grade, disodium hydrogen phosphate, sodium dihydrogen phosphate, glacial acetic acid 99%, hydrochloric acid 37%, sodium hydroxide, and Hydrogen peroxide 30% (Scharlau, Spain). Water for injection (WFI) was used in the analysis and passed through a 0.45 μm nylon membrane filter before use. Phosphate solution (1) was prepared by weighing 1.6 g of disodium hydrogen phosphate in 1000 mL of WFI. Phosphate solution (2) was prepared by weighing about 0.3 g of sodium dihydrogen phosphate in 1000 mL of WFI.

### Chromatographic system configuration

Compared with the previously conducted HPLC methods and the current analysis method, we did not use a high percentage of the organic modifier of acetonitrile, dedicated pH solution adjustment, or special chemical reagent to realize the optimum separation for the ideal system suitability achievement.

MP, 17-MPHS, and MPHS assay determination were conducted using the HPLC model HP 1100 series with variable wavelength. The current method was conducted with the RP-BDS column (250 mm × 4.6 mm × 5 μm) (Thermo Scientific). The mobile phase was prepared as WFI: glacial acetic acid: acetonitrile in a volume ratio (63:2:35) at a flow rate of 2.0 mL/min with detection wavelength at 254 nm at room temperature and injection volume 20 μL.

### Parameters of method validation

The HPLC validation method was performed according to the International Conference on Harmonization (ICH) guidelines concerning parameters including system suitability, Range of linearity, the limit of detection (LOD), the limit of quantification (LOQ), repeatability (precision), recovery and accuracy, robustness, ruggedness, the stability of the solution, specificity, and selectivity^20–22^.

### Sample preparations

#### System suitability check

System suitability was performed by injecting six replicate injections of the same sample solution which was prepared by dissolving a quantity of MP reference standard equivalent to 5 mg/100 mL of mobile phase and mixing 10 mL of this solution with a weight of MPSS working standard equivalent to 65 mg and 1 mL of each phosphate buffer solutions in 100 mL volumetric flask and complete with mobile phase to obtained a concentration about 500 µg/mL of total MP.

#### Range and linearity

The analytical approach is deemed to be linear if there is a substantial portion between the response and claimed working concentration starting at the lowest point in the tested range and increasing to the highest point with R^2^ ≥ 0.999^22–27^.

Regression linearity equation:

1 $${\text{Y }} = {\text{ a X }} \pm {\text{ b}}$$

where (Y) represents the response of the average peak area, (X) represents the claimed working concentration in (%), (a) represents the slope and (b) is the intercept of the calibration curve.

The linearity parameter was submitted using different five concentrations in the range (50–150%) of the MP working standard. The stock solutions were prepared as a quantity of MP reference standard 48.9 mg in 100 mL of the mobile phase and complete with the WFI to 1000 mL and MPSS working standard equivalent to 640 mg/100 mL in the mobile phase. Then make serial dilutions to obtain concentrations (50%, 70%, 100%, 120%, and 150%) by taking (5 mL, 7 mL, 10 mL, 12 mL, and 15 mL) from each solution of the stock solutions and complete to 100 mL with mobile phase and inject 2 replicates of each concentration.

#### Limit of detection (LOD)

It was defined as the lowest specified analyte concentration in the matrix that could be identified using the detection of the instrument. LOD concentration should not undergo the accuracy, precision, and linearity ranges every time it is injected^22–27^.

#### Limit of quantitation (LOQ)

It was defined as the lowest specified analyte concentration in the matrix that could be identified using the detection of the instrument. LOQ must undergo the accuracy, precision, and linearity ranges every time it is injected^22–27^.

LOD and LOQ could be calculated according to the slope and standard error data from the linearity of the calibration as the following:

2 $${\text{LOD }} = { 3}.{3}\upsigma /{\text{S}}$$

3 $${\text{LOQ }} = { 1}0\upsigma /{\text{S}}$$

where (σ) is the standard error of (X & Y) arrays and (S) represents the slope of the linearity calibration curve.

#### Accuracy and recovery

Both recovery and accuracy are used alternatively^28^. The measurement's accuracy is defined as the proximity of the actual concentration (measured value) to the theoretical concentration (true value)^18,20,29^.

Accuracy was implemented by the preparation of three different stock solutions of MP reference standard at 3.74, 5.49, and 6.64 mg in 100 mL mobile phase individually. Then 10 mL of each 45.7 mg, 64.8 mg, 77.4 mg/100 mL WFI of MPSS working standard individually respectively and 1 mL of each phosphate buffer solution were mixed with MP concentrations. Then injected three replicates of each concentration were to make 70%, 100%, and 120% concentrations of total MP.

Accuracy % could be estimated using the linearity equation:

4 $${\text{Accuracy }}\left( \% \right) = {\text{ Actual Conc}}. \, \left( \% \right){\text{/Theoretical Conc}}. \, \left( \% \right) \, \times { 1}00$$

#### Repeatability and precision

Repeatability was conducted using six different determinations of the 100% test concentration by dissolving about a quantity of MP reference standard equivalent to 5 mg/100 mL of mobile phase and mixing 10 mL of this solution with a weight of MPSS working standard equivalent to 65 mg and 1 mL of each phosphate buffer solutions in 100 mL volumetric flask and complete with mobile phase to obtained a concentration about 500 µg/mL of total MP^22,30^.

#### Robustness

Robustness was submitted using designed small changes including slight changes in the temperature, composition of the mobile phase, etc.^22^.

The designed small changes were conducted in a different organic solvent ratio (Acetonitrile) at (± 1%) and a flow rate (± 0.005 mL/min).

#### Ruggedness

Ruggedness was submitted using designed and major observable changes including analyst-analyst, column-column, and day-day with maintaining all of the analysis method parameters and conditions as it is without changes^23^.

#### Specificity and selectivity

The following solutions were injected individually for selectivity confirmation:

- Phosphate buffer.
- Mobile phase.
- MP reference standard.
- MP + MPHS standard.
- MP reference standard + MPHS reference standard + phosphate buffer.
- MPSS working standard.
- MP reference standard + MPSS working standard.
- MP reference standard + MPSS working standard + Phosphate buffer.
- Forced degradation studies were performed to indicate the stability indicating properties, selectivity, and specificity of the procedure using acid hydrolysis, and base H_2_O_2_ oxidation hydrolysis.
- Acid hydrolysis for MP was performed as a test under recovery test at 100% and in the final step add 10 mL of HCl [0.1 M], and it left for 30 min then complete with WFI to 100 mL.
- Base hydrolysis for MP was performed as a test under recovery test at 100% and in the final step add 10 mL of NaOH [0.1 M], and it left for 30 min then complete with WFI to 100 mL.
- H_2_O_2_ hydrolysis for MP was performed as a test under recovery test at 100% and in the final step add 10 mL of H_2_O_2_ [3.0%], and it left for 30 min then complete with WFI to 100 mL.

### Test of the validated method of the local market product of UP Pharma in Egypt

#### Methylprednisolone 1.0 g vials batch number (221160) after the constitution stability studies

The after-constitution stability study was conducted using the supplied solvent WFI at zero time, 24 h in the refrigerator at a temperature of 5 ± 3 °C.

The constituted vial was performed using 16 mL of the WFI then all of the content of the vial was transferred into a 200 mL volumetric flask. Then a dilution of 10 mL of the constituted solution (1 mg/mL) in a 100 mL volumetric flask using WFI was conducted and introduced to the HPLC for assay in a final theoretical concentration (0.5 mg/mL of MP).

### Experimental work and methods

I confirm that all methods were carried out following relevant guidelines and regulations.

## Results and discussions

### System suitability check

**Figure 2:**
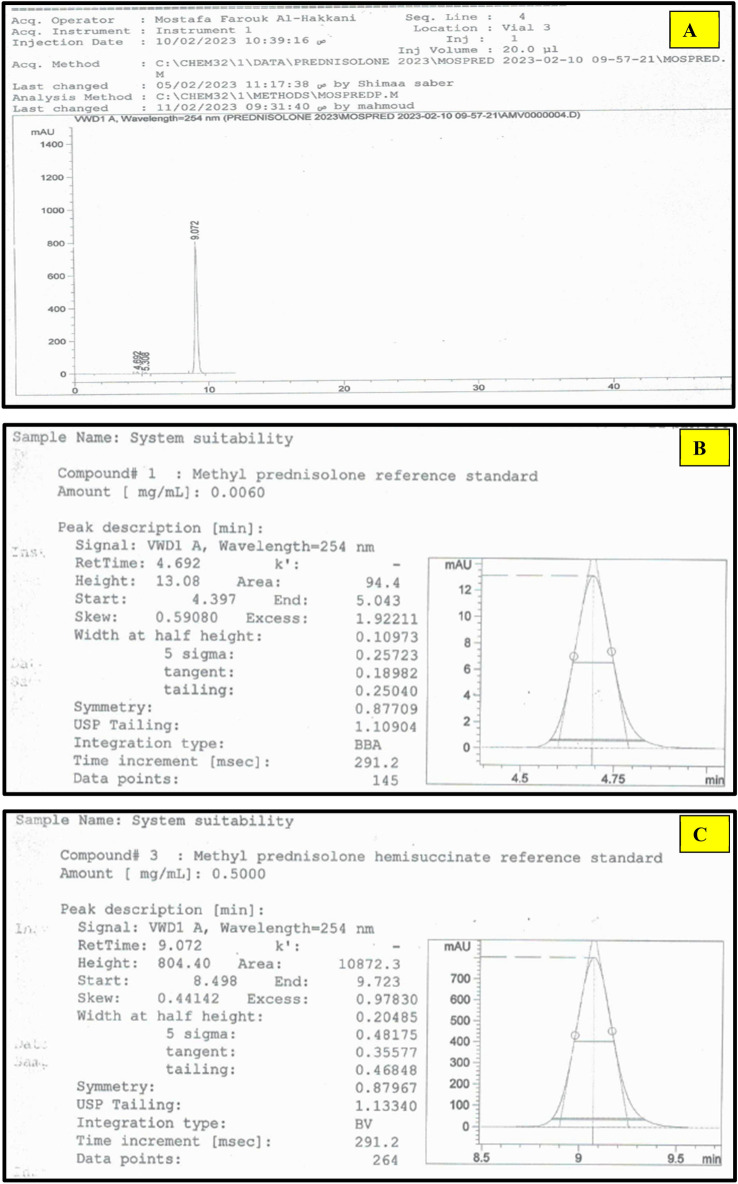
(**A**) MP, 17-MPHS, and MPHS chromatogram at an optimum HPLC parameter, USP tailing factor, and theoretical plates of (**B**) MP, (**C**) MPHS.

According to the molecular data in Table 1, the first eluted is MP according to its lower molar mass. Subsequently, 17-MPHS will elute then MPHS according to their stereochemistry where 17-MPHS has a smaller stereo shape as manifested in Fig. 1. So, the MP, 17-MPHS, and MPHS peaks appeared at retention times 4.7, 5.3, and 9.0 ± 0.2 min at the optimum parameters of the analysis method as shown in Fig. 2. The range of retention time over all the parameter changes for the three successive peaks were (4.7, 5.3, and 9.0) ± 1 min. Table 2 showed high performance for the intended analysis method where the RSD% ≤ 3.0% for 6 injections, USP tailing ≤ 2.0, and theoretical plates ≥ ^25^. So, according to the output data of the system suitability parameters, the method manifested superior validity through a wide range of retention time.

**Table 1:** Molecular data of the Mp, MPHS, and 17-MPHS.

Item	MP	MPHS	17-MPHS	MPSS
Molecular formula	C_22_H_30_O_5_	C_26_H_34_O_8_	C_26_H_34_O_8_	C_26_H_33_O_8_Na
Molar mass (g/mole)	374.47	474.54	474.54	496.54
Chemical structure	Figure 1A	Figure 1B	Figure 1C	–

**Table 2:** System suitability.

Injection/item	MP (P.A)	17-MPHS (P.A)	MPHS (P.A)	Total MPHS (P.A)
1	94.4	45.9	10,872.3	10,918.2
2	95.3	47.8	10,898.2	10,946.0
3	96.9	50.3	10,923.9	10,974.2
4	97.5	52.7	10,912.7	10,965.4
5	99.5	55.4	10,929.2	10,984.6
6	101.1	58.3	10,945.8	11,004.1
Mean	97.45	–		10,965.4
STDEV	2.52			30.2
RSD %	2.6			0.28
USP tailing	1.109	–	1.133	
Plates	10,135		10,863	
Resolution	–	3.05	13.31	

### Range and linearity

The results manifested high linearity for MP and MPHS with R^2^ = 0. 0.99981 and 0.99999 respectively at the working concentrations in the range (50–150%) as we can see in Tables 3 and 4.

**Table 3:** Range and linearity of MP.

Conc. (%)	Conc. (µg/mL)	MP (P.A)	MP mean (P.A)
50	2.445	51.2	51
		51.4	
70	3.423	75.1	76
		76.1	
100	4.890	108.5	109
		109.8	
120	5.868	133.0	133
		133.8	
150	7.335	169.2	171
		171.8	
Slope	24.227		
Intercept	− 8.110		
R^2^	0.99981

**Table 4:** Range and linearity of MPHS.

Conc. (%)	Conc. (µg/mL)	17-MPHS (P.A)	MPHS (P.A)	Total MPHS mean P.A
50	320	29.8	5553.1	5552.6
		30.8	5552.0	
70	448	46.9	7750.5	7756.1
		48.6	7761.6	
100	640	73.1	10,991.9	10,988.7
		75.7	10,985.5	
120	768	90.4	13,229.5	13,201.3
		92.5	13,173.0	
150	960	122.9	16,337.0	16,506.4
		127.2	16,675.7	
Slope	17.094			
Intercept	79.604			
R^2^	0.99999			

### LOD and LOQ

LOD and LOQ limits could be determined simply using the linearity calibration data of MP and MPHS. LOD was found to be 143.97 ng/mL and 4.49 µg/mL respectively whereas LOQ was 436.27 ng/mL and 13.61 µg/mL for MP and MPHS respectively.

### Accuracy and recovery

Tables 5 and 6 showed that the accuracy results of the tested range (70–120% from the target concentration of 100% = 500 µg/mL) were found to be within the acceptance criteria (98–102%)^20^.

**Table 5:** Accuracy and recovery of MP.

Th. conc. (%)	MP (P.A)	MP mean (P.A)	Prepared conc. (µg/mL)	Actual conc. from equation (µg/mL)	Recovery (%)
70	80.8	81	3.74	3.6943	98.8
	81.4				
	81.9				
100	121.9	123	5.49	5.4147	98.6
	123.1				
	124.1				
120	149.9	152	6.64	6.5969	99.4
	152.0				
	153.1

**Table 6:** Accuracy and recovery of MPHS.

Th. conc. (%)	MPHS (P.A)	17-MPHS (P.A)	Total MPHS mean (P.A)	Prepared conc. (µg/mL)	Actual conc. from equation (µg/mL)	Recovery (%)
70	7837	59.5	7848.2	457.0	454.465	99.4
	7836	61.1				
	7872	62.2				
100	11,103.6	95.9	11,135.2	648.0	646.753	99.8
	11,145.2	98.4				
	11,156.8	100.6				
120	13,321.7	122.7	13,293.1	774.0	772.988	99.9
	13,289.3	126.1				
	13,268.2	128.8				

### Repeatability and precision

The RSD% of peak areas was used for judgment on the repeatability of the analyte using six different preparations at the same target (500 µg/mL of MP) concentration. It was found to be 1.9% and 0.28% within intra-precision and 1.8% and 0.43% at the inter-precision for the MP and MPHS respectively over 2 days as it demanded in repeatability requirements RSD% < 2.0%^31^ as Table 7 manifested.

**Table 7:** Repeatability of MP and MPHS.

Item	MP (P.A)	Wt (mg)	17-MPHS (P.A)	MPHS (P.A)	Total MPHS (P.A)	Wt (mg)
Day-1						
1	130.10	5.79	114.6	10,993.7	11,108.3	62.9
2	136.90	6.11	117.6	10,975.5	11,093.1	62.8
3	137.00	6.11	124.2	10,932.3	11,056.5	62.6
4	134.90	6.01	122.8	10,908.3	11,031.1	62.3
5	135.30	6.04	124.1	10,930.6	11,054.7	62.6
6	135.50	6.28	132.7	10,968.2	11,100.9	62.9
Mean	134.95	–			11,074.1	
STDEV	2.53				30.93	
RSD (%)	1.9				0.28	
Day-2						
1	107.00	4.90	67.0	11,035.3	11,102.3	63.3
2	109.60	4.91	71.8	11,109.9	11,181.7	63.4
3	108.80	4.89	71.1	11,041.5	11,112.6	63.2
4	111.80	4.93	75.6	10,995.2	11,070.8	63.2
5	110.90	4.93	77.4	11,076	11,153.4	63.2
6	107.30	4.84	78.8	11,111.8	11,190.6	63.4
Mean	109.2	–			11,135.2	
STDEV	1.92				47.55	
RSD (%)	1.8				0.43	

### Robustness

The results of conscious small changes included a flow rate ± 0.005 mL/min and acetonitrile (± 1%) was determined using RDS%. The RSD% was found to be ≤ 3% in all cases as shown in Tables 8 and 9. It is clear for man there is a reverse proportion between the retention time and the ratio of the organic modifier of the acetonitrile^28^. Where the retention time increases by decreasing the organic ratio and vice versa. This assures the principle chromatographic rule “likes to dissolve likes or likes attract likes”^24–27^.

**Table 8:** Flow rate change effect on MP and MPHS.

Item	MP (P.A)	17-MPHS (P.A)	MPHS (P.A)	Total MPHS (P.A)
2.0 mL/min				
1	94.4	45.9	10,872.3	10,918.2
2	95.3	47.8	10,898.2	10,946
3	96.9	50.3	10,923.9	10,974.2
4	97.5	52.7	10,912.7	10,965.4
5	99.5	55.4	10,929.2	10,984.6
6	101.1	58.3	10,945.8	11,004.1
Mean	97.45	–		10,965.4
STDEV	2.52			30.2
RSD (%)	2.6			0.28
USP tailing	1.109	–	1.133	–
Plates	10,135		10,863	
Resolution	3.05			
2.005 mL/min				
1	120.80	91.3	11,062.7	11,154.0
2	122.30	94	11,055.2	11,149.2
3	124.10	96.8	11,053.6	11,150.4
Mean	122.4	–		11,151.2
STDEV	1.65			2.50
RSD (%)	1.3			0.02
USP tailing	1.1	–	1.1	–
Plates	8317.0		8632.0	
Resolution	2.75			
1.995 mL/min				
1	121.40	95.9	11,167.2	11,263.1
2	122.90	98.7	11,192.8	11,291.5
3	124.20	100.9	11,165.2	11,266.1
Mean	122.8333	–		11,273.6
STDEV	1.40			15.60
RSD (%)	1.1			0.14
USP tailing	1.0	–	1.1	–
Plates	8723.0		8519.0	
Resolution	2.73

**Table 9:** Acetonitrile rate change effect on MP and MPHS.

Item	MP (P.A)	17-MPHS (P.A)	MPHS (P.A)	Total MPHS (P.A)
Acetonitrile (100%)				
1	94.4	45.9	10,872.3	10,918.2
2	95.3	47.8	10,898.2	10,946.0
3	96.9	50.3	10,923.9	10,974.2
4	97.5	52.7	10,912.7	10,965.4
5	99.5	55.4	10,929.2	10,984.6
6	101.1	58.3	10,945.8	11,004.1
Mean	97.45	–		10,965.4
STDEV	2.52			30.2
RSD (%)	2.6			0.28
USP tailing	1.109	–	1.133	–
Plates	10,135		10,863	
Resolution	3.05			
Acetonitrile (99%)				
1	167.10	186.9	11,031.4	11,218.3
2	168.30	188.8	11,023.5	11,212.3
3	169.40	191.1	11,025.8	11,216.9
Mean	168.3	–		11,215.8
STDEV	1.15			3.1
RSD (%)	0.68			0.03
USP tailing	1.093	–	1.091	–
Plates	9927.0		10,543.0	
Resolution	3.04			
Acetonitrile (101%)				
1	186.50	196.1	10,939.4	11,135.5
2	188.70	199.5	10,961.2	11,160.7
3	190.80	202.5	10,956.5	11,159
Mean	188.6667	–		11,151.7
STDEV	2.15			14.08
RSD (%)	1.1			0.13
USP tailing	1.099	–	1.096	–
Plates	9965.0		10,628.0	
Resolution	3.04			

### Ruggedness

The results of conscious major and observable changes include analyst-analyst, day-day, and column-column. Data was presented as shown in Tables 10, 11, 12. RSD% was found to be < 3% in all cases^23^.

**Table 10:** Analyst-analyst effect on MP and MPHS.

Item	MP (P.A)	17-MPHS (P.A)	MPHS (P.A)	Total MPHS (P.A)
Analyst-1				
1	94.4	45.9	10,872.3	10,918.2
2	95.3	47.8	10,898.2	10,946
3	96.9	50.3	10,923.9	10,974.2
4	97.5	52.7	10,912.7	10,965.4
5	99.5	55.4	10,929.2	10,984.6
6	101.1	58.3	10,945.8	11,004.1
Mean	97.45	–		10,965.4
STDEV	2.52			30.2
RSD (%)	2.6			0.28
USP tailing	1.109	–	1.133	–
Plates	10,135		10,863	
Resolution	3.05			
Analyst-2				
1	149.50	142.2	10,942.6	11,084.8
2	151.40	145.1	10,925.6	11,070.7
3	153.50	148.4	10,918	11,066.4
Mean	151.4667	–		11,074.0
STDEV	2.00			9.63
RSD (%)	1.3			0.09
USP tailing	1.099	–	1.099	–
Plates	10,136		10,824	
Resolution	3.05

**Table 11:** Column-column effect on MP and MPHS.

Item	MP (P.A)	17-MPHS (P.A)	MPHS (P.A)	Total MPHS (P.A)
Column-1				
1	94.4	45.9	10,872.3	10,918.2
2	95.3	47.8	10,898.2	10,946.0
3	96.9	50.3	10,923.9	10,974.2
4	97.5	52.7	10,912.7	10,965.4
5	99.5	55.4	10,929.2	10,984.6
6	101.1	58.3	10,945.8	11,004.1
Mean	97.45	–		10,965.4
STDEV	2.52			30.2
RSD (%)	2.6			0.28
USP tailing	1.109	–	1.133	–
Plates	10,135		10,863	
Resolution	3.05			
Column-2				
1	179.00	203.9	10,999.4	11,203.3
2	179.70	205.6	10,985.5	11,191.1
3	181.70	209.3	11,005.1	11,214.4
Mean	180.13	–		11,151.2
STDEV	1.40			2.50
RSD (%)	0.8			0.02
USP tailing	1.03	–	1.07	–
Plates	10,878		10,460	
Resolution	3.03

**Table 12:** Day-day effect on MP and MPHS.

Item	MP (P.A)	17-MPHS (P.A)	MPHS (P.A)	Total MPHS (P.A)
Day-1				
1	94.4	45.9	10,872.3	10,918.2
2	95.3	47.8	10,898.2	10,946.0
3	96.9	50.3	10,923.9	10,974.2
4	97.5	52.7	10,912.7	10,965.4
5	99.5	55.4	10,929.2	10,984.6
6	101.1	58.3	10,945.8	11,004.1
Mean	97.45			10,965.4
STDEV	2.52			30.2
RSD (%)	2.6			0.28
USP tailing	1.109		1.133	
Plates	10,135		10,863	
Resolution	3.05			
Day-2				
1	100.9	54	11,055.3	11,109.3
2	102.4	56.2	11,129.5	11,185.7
3	103.8	58.7	11,037.6	11,096.3
4	105.2	61.1	11,068.4	11,129.5
5	103.1	63.3	11,057	11,120.3
6	105.4	65.6	11,005.1	11,070.7
Mean	103.4667			11,118.6
STDEV	1.72			38.7
RSD (%)	1.7			0.35
USP tailing	1.042		1.053	
Plates	8459		8835	
Resolution	2.83			

### Specificity and selectivity

The current method supplied us with highly specific data about the resolution and separation performance of the adjacent co-eluted peaks for the MP, 17-MPHS, and MPHS principal peaks. The smallest resolution was found to be 2.54 in the case of MP + MPSS + Buffer as tabulated in Table 13.

**Table 13:** Specificity and selectivity investigation.

Item	Resolution
Phosphate buffer	No peaks response
Mobile phase	No peaks response
MP	One peak only
MP + MPHS	13.53
MP + MPHS + buffer	13.47
MPSS	10.94
MP + MPSS	2.56
MP + MPSS + buffer	2.54
Test + HCl [0.1 M] degradation	It gives a white precipitate
Test + NaOH [0.1 M] degradation	3.06 without any presence of new peaks
Test + H_2_O_2_ degradation	3.07 without any presence of new peaks

### Test of the validated method of the local market product of UP Pharma in Egypt

#### Methylprednisolone 1.0 g vials batch number (221,160) after the constitution stability studies

The tabulated results of the stability studies in Table 14 confirmed the stability and validity of the use of the MP solutions after constitution using WFI, sodium chloride 0.9% wt/v, and glucose 5%wt/v solutions at 5 ± 3 °C for 24 h. Where the assay % was found to be within the acceptance criteria (90–110%) of the stated amount and did not exceed 2.0% from the starting assay at zero time. Also, the results manifested that the method did not affect the composition of the different initiators of the solvent on the retention time over the study.

**Table 14:** After the constitution of methylprednisolone 1.0 g vials using WFI, Dextrose 5%, and NaCl 0.9%.

After the constitution using WFI								
Item	Zero time				24 h @2–8 °C			
	MP (P.A)	17-MPHS (P.A)	MPHS (P.A)	Total MPHST (P.A)	MP (P.A)	17-MPHS (P.A)	MPHS (P.A)	Total MPHST (P.A)
1	130.6	108.9	11,083.8	11,192.7	110.1	57.8	11,238.7	11,296.5
2	132.2	111.5	11,078.4	11,189.9	110.3	57.4	11,286.4	11,343.8
3	134.2	115.4	11,101.9	11,217.3	112.1	60.8	11,380.4	11,441.2
Mean (P.A)	132.33	111.93	11,088.03	11,199.97	110.83	58.67	11,301.83	11,360.50
Assay (%)	1.18	1.0	101.6	102.6	0.98	0.5	103.6	104.1
Total assay as MP (%)	103.8				105.1			

After the constitution using dextrose 5%								
Item	Zero time				24 h @2–8 °C			
	MP (P.A)	17-MPHS (P.A)	MPHS P.A	MP (P.A)	17-MPHS (P.A)	17-MPHST	MP (P.A)	17-MPHS (P.A)
1	126.1	106.5	10,960	11,066.5	127.1	74	11,122.2	11,196.2
2	127.1	109.1	10,920.2	11,029.3	128.2	76.1	11,117.4	11,193.5
3	128.7	111.9	10,916.4	11,028.3	129.2	78.1	11,128	11,206.1
Mean (P.A)	127.3	109.17	10,932.2	11,041.37	128.16667	76.07	11,122.533	11,198.60
Assay (%)	1.13	1.0	100.2	101.2	1.14	0.7	101.9	102.6
Total assay as MP (%)	102.3				103.7			

After the constitution using NaCl 0.9%								
Item	Zero time				24 h @2–8 °C			
	MP (P.A)	17-MPHS (P.A)	MPHS P.A	MPT (P.A)	17-MPHS (P.A)	17-MPHS	MP (P.A)	17-MPHS (P.A)
1	117.4	100.3	10,959.5	11,059.8	131.7	81.7	11,142.2	11,223.9
2	118.6	102.7	10,985.4	11,088.1	131	81.7	11,067.3	11,149
3	119.5	105	10,938.4	11,043.4	132	83.7	11,059.9	11,143.6
Mean (P.A)	118.5	102.67	10,961.1	11,063.77	131.56667	82.37	11,089.8	11,172.17
Assay (%)	1.05	0.9	100.4	101.4	1.17	0.8	101.6	102.4
Total assay as MP (%)	102.4				103.5			

## Conclusions

The validated method was evaluated and it was found to be sensitive to detecting the low concentration of the free Methyl Prednisolone and Methyl Prednisolone hemi succinate at LOD 143.97 ng/mL and 4.49 µg/mL respectively with LOQ 436.27 ng/mL and 13.61 µg/mL. Also, the method was found to be accurate from concentration level 70 µg/mL to 120 µg/mL with high accuracy for free Methyl Prednisolone and Methyl Prednisolone hemi succinate (98.8–99.4%) and (99.4–99.9%) respectively, precise and repeatable over two days with intra precision and inter precision. The linearity of the method was conducted in the range 250 µg/mL to 750 µg/mL with excellent regression coefficient R^2^ = 0.9998–0.99999 for free Methyl Prednisolone and Methyl Prednisolone hemi succinate respectively. The method's robustness was evaluated through minor deliberated changes in implementation as different flow rates, different mobile phase compositions, different days, and analysts. It proved its high capability to achieve the requirement of the chromatographic system suitability as the following, theoretical plates and column efficiency ≥ 2000, USP tailing at ≤ 2.0. Finally, the selectivity and specificity of the current method were confirmed by realizing the minimum resolution between the Methyl Prednisolone principal peak and the most adjacent related impurity peak at 2.54. The validated method proved its performance capability in the separation of the Methyl Prednisolone principal peak from any other appearance-forced degradation peaks.

## Data Availability

All data generated or analyzed during this study are included in this article.

## Abbreviations

MP: Methyl prednisolone reference standard
MPHS: Methyl prednisolone hemisuccinate reference standard
MPSS: Methyl prednisolone sodium succinate working standard
Conc: Concentration
HPLC: High-performance liquid chromatography
RP- HPLC: Reversed phase-high-performance liquid chromatography
UV: Ultraviolet
LOD: Limit of detection
LOQ: Limit of quantitation
P. A: Peak area
RSD: Relative standard deviation
STDEV: Standard deviation
USP: United States Pharmacopeia
WFI: Water for injection
